# Remotely prescribed, monitored, and tailored home-based gait-and-balance exergaming using augmented reality glasses: a clinical feasibility study in people with Parkinson’s disease

**DOI:** 10.3389/fneur.2024.1373740

**Published:** 2024-05-30

**Authors:** Lotte E. S. Hardeman, Daphne J. Geerse, Eva M. Hoogendoorn, Jorik Nonnekes, Melvyn Roerdink

**Affiliations:** ^1^Department of Human Movement Sciences, Faculty of Behavioural and Movement Sciences, Vrije Universiteit Amsterdam, Amsterdam Movement Sciences, Amsterdam, Netherlands; ^2^Radboud University Medical Centre, Donders Institute for Brain, Cognition and Behaviour, Department of Rehabilitation, Centre of Expertise for Parkinson and Movement Disorders, Nijmegen, Netherlands; ^3^Department of Rehabilitation, Sint Maartenskliniek, Nijmegen, Netherlands

**Keywords:** Parkinson’s disease, augmented reality, gait, balance, walking adaptability, exergaming, digital therapeutics

## Abstract

**Background:**

Exergaming has the potential to increase adherence to exercise through play, individually tailored training, and (online) remote monitoring. Reality Digital Therapeutics (Reality DTx^®^) is a digital therapeutic software platform for augmented reality (AR) glasses that enables a home-based gait-and-balance exergaming intervention specifically designed for people with Parkinson’s disease (pwPD).

**Objective:**

The primary objective was to evaluate the feasibility and potential efficacy of Reality DTx^®^ AR exergaming intervention for improving gait, balance, and walking-adaptability fall-risk indicators. The secondary objective was to evaluate the potential superiority of AR glasses [Magic Leap 2 (ML2) vs. HoloLens 2 (HL2)].

**Methods:**

This waitlist-controlled clinical feasibility study comprised three laboratory visits (baseline; pre-intervention; and post-intervention), a home visit, and a 6-week AR exergaming intervention. Five complementary gait-and-balance exergames were remotely prescribed (default five sessions/week of 30 active minutes/session), monitored, and tailored. Feasibility was assessed in terms of safety, adherence, and user experience. During laboratory visits, gait-and-balance capacity was assessed using standard clinical gait-and-balance tests and advanced walking-adaptability fall-risk assessments.

**Results:**

In total, 24 pwPD participated. No falls and four near falls were reported. Session adherence was 104%. The User Experience Questionnaire scores for Reality DTx^®^ ranged from above average to excellent, with superior scores for HL2 over ML2 for Perspicuity and Dependability. Intervention effects were observed for the Timed Up and Go test (albeit small), the Five Times Sit to Stand test, and walking speed. Walking-adaptability fall-risk indicators all improved post-intervention.

**Conclusion:**

Reality DTx^®^ is a safe, adherable, usable, well-accepted, and potentially effective intervention in pwPD. These promising results warrant future randomized controlled trials on the (cost-)effectiveness of home-based AR exergaming interventions for improving gait, balance, and fall risk.

**Clinical trial registration:**

ClinicalTrials.gov, identifier NCT05605249.

## 1 Introduction

People with Parkinson’s disease (pwPD) experience a wide range of gait-and-balance impairments, significantly affecting functional mobility and quality of life (1–7). Clinical (physiotherapy) guidelines stress the central role of exercise in the disease management of motor and non-motor symptoms (8–12). Exercise is defined as a planned, structured, repetitive, and purposeful physical activity to maintain one or more components of physical fitness (7). Despite increasing recognition of the importance of exercise in disease management, adherence to exercise remains challenging (13).

In this clinical feasibility study, we evaluated a 6-week remotely prescribed, monitored, and tailored home-based augmented reality (AR) exergaming (i.e., “exercise” and “gaming”) intervention (Reality DTx®) designed for state-of-the-art AR glasses [Magic Leap 2 (ML2); Microsoft HoloLens 2 (HL2)]. Our main therapeutic goal with this digital therapeutics program Reality DTx® was to improve gait and balance, including walking adaptability, in pwPD through gamified rehabilitation exercises. Moreover, Reality DTx® aims to increase the dose and adherence to exercise by making exercise more accessible (at home, at any time) and enjoyable, thereby potentially increasing the number of (unsupervised) rehabilitation exercise hours.

Reality DTx® is designed to accommodate individually tailored exercise [following FITT principles; frequency, intensity, type, and time (7)], to monitor exercise remotely (in terms of adherence and performance), and to motivate the user through gamification and feedback, all important aspects for delivering a progressive-but-achievable intervention. To date, research on home-based exergaming interventions for pwPD primarily focused on non-immersive devices (e.g., Xbox Kinect or Nintendo Wii), showing promise in providing a safe and effective intervention for improving balance, mobility, and gait (14–18). The effectiveness of the in-clinic use of such non-immersive exergaming interventions is considered at least equivalent to traditional physiotherapy and strengthens the effects of traditional physiotherapy when combined (19–22). Recognition for the use of AR head-mounted displays for in-home rehabilitation, like the ones used in the present study, is increasing (23, 24).

The primary objective of this pre-registered waitlist-controlled clinical feasibility trial was to evaluate feasibility (in terms of safety, adherence, and user experience) and potential efficacy for improving clinical gait-and-balance test scores and laboratory-based targeted walking-adaptability fall-risk indicators. The secondary objective was to evaluate the potential superiority of state-of-the-art AR glasses (i.e., ML2 vs. HL2) for delivering Reality DTx®.

## 2 Methods

Here, we summarize the methods used in this study. A detailed study protocol was pre-registered (25), while (minor) changes thereto are specified below.

### 2.1 Participants

Participants were eligible to participate if diagnosed with PD according to the UK PD Brain Bank criteria [Hoehn and Yahr Scale (HY) stage 2–4] and experienced bothersome gait and/or balance impairments based on self-report. Participants were excluded if there was a sign of inability to comply with protocol, additional neurological diseases and/or orthopedic problems seriously interfering with gait-and-balance function, insufficient physical capacity or cognitive and/or communicative inability to understand instructions and participate in the tests (as observed by the researchers), visual or hearing impairments (after corrective aids), severe visual hallucinations or illusions, inability to walk independently for 30 min, and no stable dosages of dopaminergic medication. There were no restrictions to usual care. Eligibility criteria were checked through telephone screening before enrollment and again during the baseline laboratory assessment.

Ethical approval was obtained from the accredited Medical Research Ethics Committees United, The Netherlands (R22.076, NL82441.100.22, under the title “CueX: a gamified gait-and-balance exercise intervention for augmented reality glasses to improve Parkinsonian gait”), and the research was carried out in accordance with the principles laid down by the Declaration of Helsinki. Participants provided written informed consent obtained by researchers LH, DG, or EH before participating in this study.

### 2.2 Trial design, intervention, and procedure

**Figure 1:**
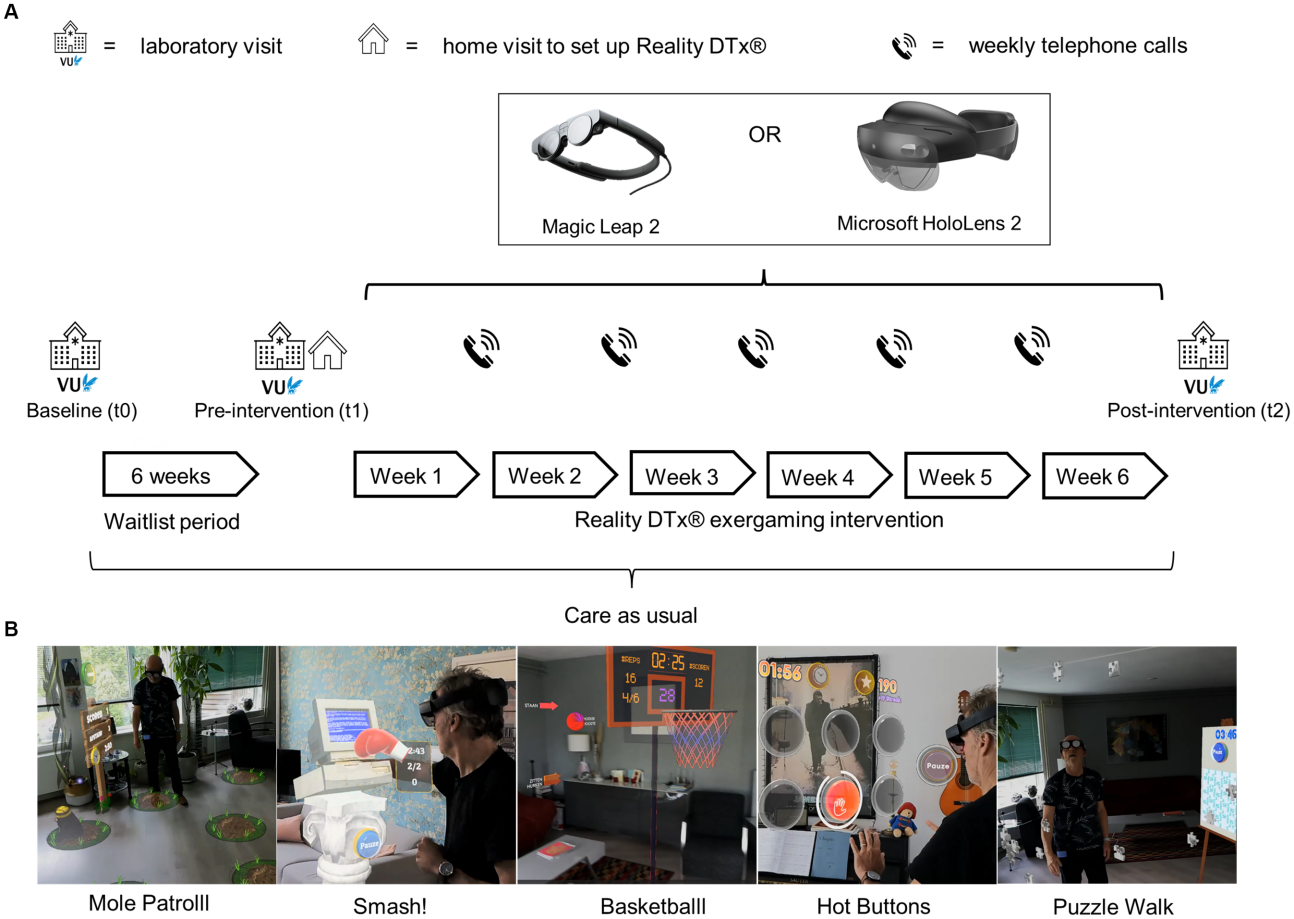
**(A)** Overview of the study design and procedure, with **(B)** images of the five exergames of Reality DTx^®^.

**Figure 2:**
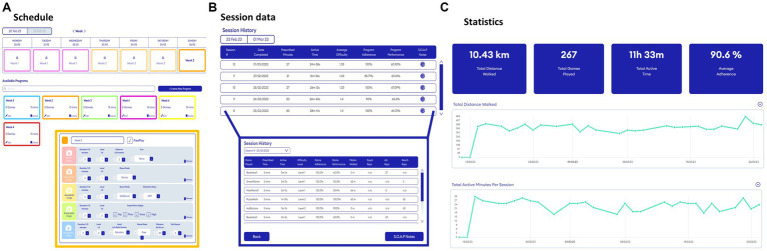
Snapshots of the web portal to remotely prescribe **(A)** and monitor **(B,C)** gait-and-balance exergames. Please see Supplementary Table S1 for a description of all adjustable Reality DTx^®^ gait-and-balance exergaming elements per game.

This waitlist-controlled feasibility trial (Figure 1) comprised:

- three laboratory assessments (baseline [t0], pre-intervention [t1], and post-intervention [t2]), see 26.
- a 6-week waitlist period (between t0 and t1) to evaluate effects, if any, of usual care,
- a home visit to set up Reality DTx® for independent but remotely monitored use,
- a 6-week home-based Reality DTx® intervention period with weekly telephone calls in addition to usual care. Reality DTx® is an AR software application (registered as a UKCA, FDA, and CE-marked medical device) for delivering a home-based gait-and-balance exergaming rehabilitation program. Reality DTx® is remotely prescribed and monitored through a web portal (Figure 2) and delivered through state-of-the-art ML2 or HL2 AR glasses, randomized over participants to evaluate the potential superiority of AR glasses (Figure 1),
- The Reality DTx® intervention comprises five complementary gait-and-balance exergames, developed in collaboration with Strolll Limited (Figure 1; see Supplementary material for a Video and Supplementary Table S1 for a detailed game description). Participants were initially instructed to use Reality DTx® for 30 active minutes/day (in one session or divided over the day in ‘exercise snacks’) for 5 days/week but were allowed to train more. Reality DTx^®^ was intended to be a progressive-but-achievable intervention. Hence, it was personalized (i.e., in terms of frequency, type, difficulty, duration, or mode of the exergames) and updated on a weekly basis, with shared decision-making among participants and trial managers using feedback from weekly telephone calls and remotely monitored adherence and performance data from the web portal (Figure 2) as input.

### 2.3 Outcomes

**Figure 3:**
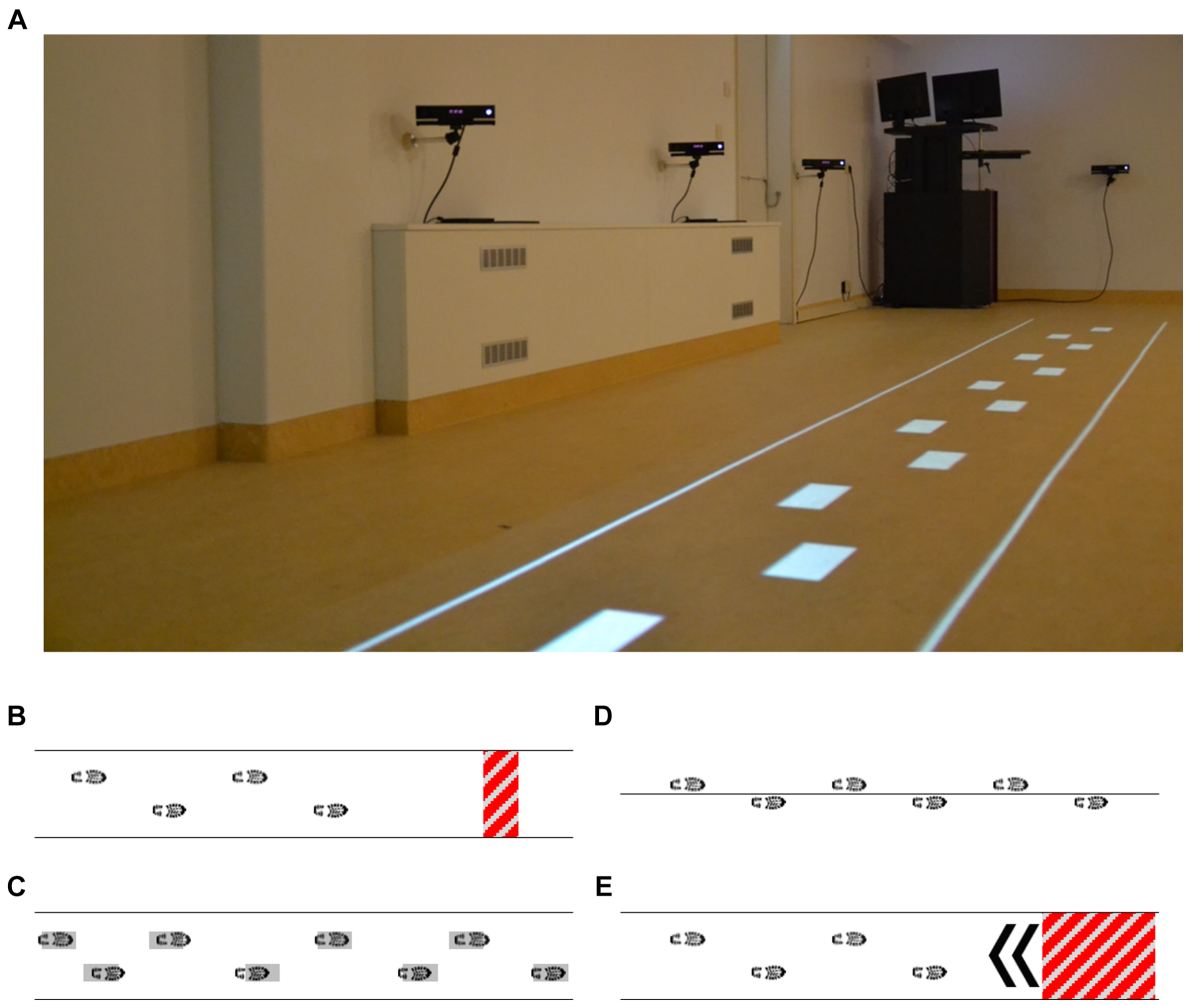
Visual representation of the interactive walkway **(A)** used for a targeted fall-risk assessment, including gait (instrumented 10-m walk test) and adaptive gait [augmented obstacle-avoidance **(B)**, goal-directed stepping **(C)**, tandem walking **(D)**, and half-turn **(E)** tasks] assessments.

Various complementary outcomes of potential efficacy for improving gait and balance were evaluated in the laboratory (t0, t1, and t2), using clinical gait-and-balance tests and adaptive-walking tasks like obstacle avoidance with the Interactive Walkway (Figure 3), which allowed for more in-depth targeted fall-risk assessment. Complementary feasibility outcomes were derived from the web portal (adherence and performance scores), telephone calls (safety and technical issues), and online questionnaires (acceptability and user experience) during (t1-t2) or after (>t2) the intervention as specified in Supplementary material S2 and detailed in the pre-registration (25).

### 2.4 Statistical analyses

#### 2.4.1 Planned analyses

Independent-samples *t*-tests (or their non-parametric equivalents) were used to evaluate safety and user experience between groups (ML2 vs. HL2). Adherence was analyzed with a 2 (between-subjects factor Group: ML2, HL2) × 6 (within-subject factor Week: 1 to 6) mixed ANOVAs, with a polynomial contrast analysis to evaluate a trend in adherence between weeks. Potential efficacy outcomes were subjected to 2 × 3 mixed ANOVAs with the between-subjects factor Group and the within-subject factor Time (three levels: t0, t1, and t2). For the main effects of Time, the first and second reverse Helmert contrasts were used to evaluate waitlist and intervention effects, respectively. Data analysis was performed in JASP (27), with significance set at 0.05 and effect size reported as partial-eta squared. Missing data, due to, for example, technical issues and missed medication dose, were excluded from the analysis. Conditions for parametric testing were checked for all analyses. If violated, appropriate non-parametric tests were used. Bayesian hypothesis testing was performed to quantify the likelihood of support for the alternative hypothesis over the null [BF_10_-values between 1 and 3, between 3 and 10, and above 10 reflect, respectively, anecdotal, moderate, and strong evidence for the alternative hypothesis (28)].

#### 2.4.2 Exploratory analyses (not specified in the pre-registration)

Reality DTx® was intended as a progressive-but-achievable rehabilitation intervention, where exergame-level settings can be tailored to the varying abilities and progression rates of participants. To evaluate this progressive-but-achievable nature, we compared each game on: (i) Reality DTx® exergame-level settings (5 levels, specified in Supplementary Table S1) over the 6-week intervention using a chi-square test for independence (an increase in game-play levels was expected over weeks) and (ii) the game-play performance scores over the 6-week intervention using a mixed ANOVA (high-but-submaximal scores were expected, without differences over weeks).

## 3 Results

### 3.1 Participant inclusion, characteristics, and dropouts

**Figure 4:**
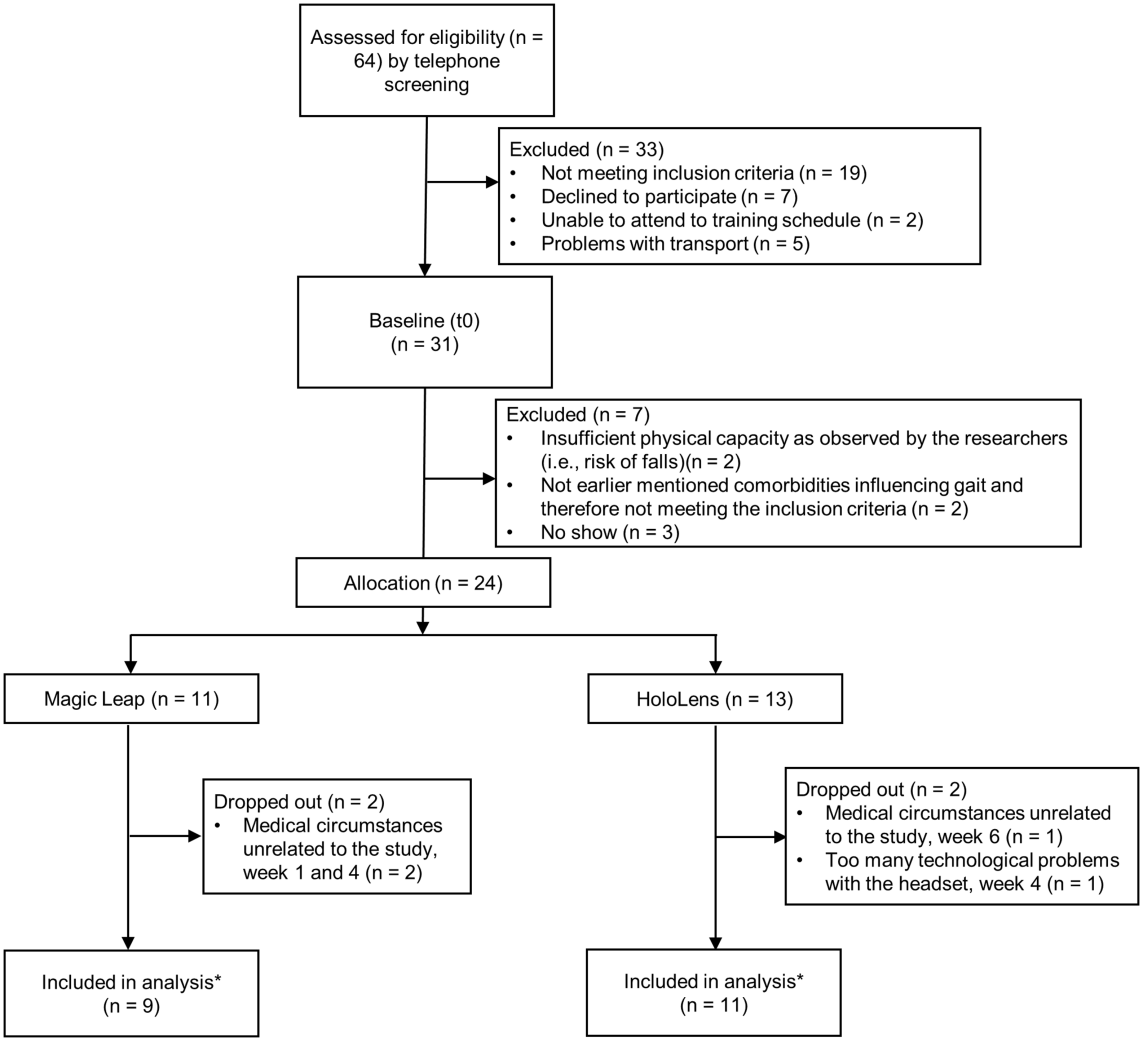
Flow diagram of the 24 study participants. Note: *One participant changed medication dose (700 to 800 mg levodopa/carbidopa) in the waitlist-control period (three weeks before pre-intervention measures, t1) and was not excluded because we consider this small change in medication acceptable as part of this feasibility study.

In total, 24 of the 31 participants scheduled for a baseline assessment (t0) started the Reality DTx® intervention (Figure 4). There were three no-shows. Two persons were excluded for ‘*insufficient physical capacity as observed by the researchers*’ (i.e., their fall risk during unsupervised home-based exergaming was deemed too high, both were classified as HY3, were freezers [New Freezing of Gait Questionnaire (NFOGQ) scores of 13/28 and 24/28], and reported considerably higher fall rates [1–2 falls/week] than the other participants [max 10 falls/year; Table 1]). Two persons were excluded for ‘*comorbidities influencing gait*’ [i.e., cerebral vascular accident and weakness in L5 musculature (dorsiflexors and hip abductors)]. Baseline characteristics did not differ for the 24 participants randomized to the ML2 (*n* = 11) and the HL2 (*n* = 13) AR glasses groups (Table 1, please see section 3.2.4 for a clarification on the difference in number of participants per group). Four of these 24 participants dropped out of the study after t1, yielding a dropout rate of 16.7% (Figure 4). Dropouts who trained for at least 3 weeks (i.e., three of four) were included in the feasibility analyses of safety and were administered the user experience questionnaires because we did not want to limit these analyses to only those participants who finished the intervention. That is, to minimize bias and learn from dropouts to optimize the intervention, we included a total of 23 participants in the feasibility analyses.

**Table 1:** Baseline participant characteristics did not differ between the HL2 and ML2 groups.

	ML2 (*n* = 11)	HL2 (*n* = 13)	Statistic
Age (years)	69.8 [53–82]	64 [51–74]	*t*(22) *=* −1.639, *p =* 0.116, BF_10_ = 0.966
Sex	8M, 3F	9M, 4F	*X*^2^(1) = 0.035, *p* = 0.851, BF_10_ = 0.509
Disease duration (years)	9 [1–15]	7 [1–20]	*t*(22) *=* −0.949, *p =* 0.353, BF_10_ = 0.519
Modified HY	2 (45.5%), 2.5 (54.5%)	2 (69.2%), 2.5 (30.8%)	*X*^2^(1) = 1.386, *p* = 0.239, BF_10_ = 0.900
MoCA score	27 [19–30]	26 [18–29]	*U* = 41.000, *p =* 0.078, BF_10_ = 1.109
LEDD (max. mg/day)	814 [150–1738]	866 [125–2,400]	*t*(22) *=* 0.429, *p =* 0.672, BF_10_ = 0.411
History of falls (per year)	2.5 [0–10]	2.6 [0–10]	*U* = 70.500, *p =* 0.976, BF_10_ = 0.372
Number of freezers	7	5	*X*^2^(1) = 1.510, *p* = 0.219, BF_10_ = 0.942
MDS-UPDRS (total score)	69 [50–79]	58 [34–78]	*t*(22) *=* −1.904, *p =* 0.070, BF_10_ = 2.092
PASE	117.7 [45.0–180.0]	128.0 [40.0–246.4]	*t*(22) *=* 0.404, *p =* 0.690, BF_10_ = 0.397

Data are mean [range]. Disease duration (years) = time since diagnosis. LEDD, levodopa equivalent daily dose; Modified HY, the Modified Hoehn and Yahr Scale; MoCA, the Montreal Cognitive Assessment; MDS-UPDRS, the MDS-Unified Parkinson’s Disease Rating Scale; PASE, the Physical Activity Scale for the Elderly.

### 3.2 Feasibility

#### 3.2.1 Safety

There were no serious adverse events during the Reality DTx® intervention. Table 2 shows the number of reported adverse events per week. There were no falls and four near falls reported by three unique participants; nine participants experienced 15 dizziness events, one participant experienced a headache twice, none reported eyestrain, and 11 participants reported 27 experiences of other adverse events, such as re-occurring prior injuries (e.g., low back or shoulder pain, the latter due to fatigue, and pinched-nerve complaints), aggravated existing PD-related (e.g., dystonia and dyskinesia), or comorbid (e.g., COPD and fibromyalgia) symptoms, often reported by the same participant over multiple training weeks. There were no group effects (ML2 vs. HL2).

**Table 2:** Adverse events.

	Number of experienced adverse events per week						Total number of reported adverse events/total number of training weeks		Total number of unique participants reporting an adverse event/total number of participants	
	Week 1	Week 2	Week 3	Week 4	Week 5	Week 6	HL2	ML2	HL2	ML2
Falls	0/23	0/23	0/23	0/21	0/21	0/20	0/74	0/57	0/23	0/23
Near falls	1/23	0/23	1/23	0/21	2/21	0/20	3/74	1/57	2/23	1/23
Dizziness	5/23	4/23	2/23	1/21	2/21	1/20	11/74	4/57	6/23	3/23
Headache	1/23	0/23	1/23	0/21	0/21	0/20	2/74	0/57	1/23	0/23
Eyestrain	0/23	0/23	0/23	0/21	0/21	0/20	0/74	0/57	0/23	0/23
Other	3/23	1/23	7/23	5/21	7/21	4/20	23/74	4/57	8/23	3/23

#### 3.2.2 Adherence

**Figure 5:**
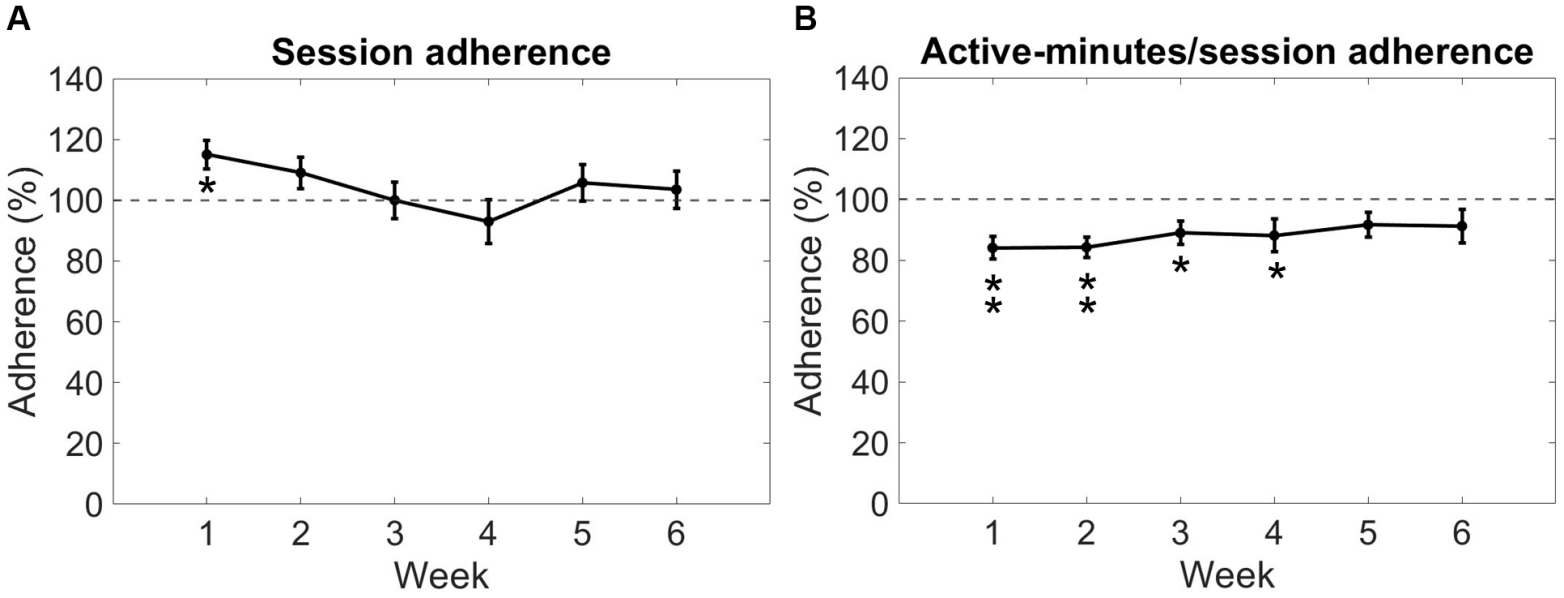
Reality DTx® adherence over weeks in terms of session adherence **(A)** and active minutes/session adherence **(B)**. Error bars represent the standard error of the mean. *p < 0.01, **p < 0.001.

For the 20 participants completing the Reality DTx® intervention, a total of 606 Reality DTx® sessions were performed, while 583 sessions were prescribed, amounting to an overall 104% session adherence. Session adherence varied significantly over weeks (*F*(5,90) = 3.438, *p* = 0.007, *η_p_^2^* = 0.160, *BF_10_* = 6.789, with a significant quadratic contrast *t*(19) = 3.441, *p* = 0.003; Figure 5A), without main or interaction effects involving groups. One-sample *t*-tests against 100% only revealed a significant difference for week 1 (*Z* = 102.500, *p* = 0.014), wherein participants performed more sessions than prescribed (Figure 5A). Participants, on average, walked 9,989 ± 3,889meters, performed 1,633 ± 834 sit-to-stand/squat movements, performed 14,218 ± 5,400 functional reaches, and completed 790 ± 246 active exercise minutes, amounting to 88% active minutes/session adherence, which did not vary significantly over weeks (*F*(3.45,62.04) = 0.765, *p* = 0.535, *η_p_^2^* = 0.041, *BF_10_* = 0.076). One-sample *t*-tests against 100% revealed that participants performed fewer than prescribed active minutes/session in weeks 1, 2, 3, and 4 (*t*(19) = −4.332, *p* < 0.001, *t*(19) = −4.808, *p* < 0.001, *t*(19) = −2.888, *p* = 0.009 and *Z* = 28.000, *p* = 0.007, respectively; Figure 5B).

#### 3.2.3 Progressive-but-achievable intervention

**Figure 6:**
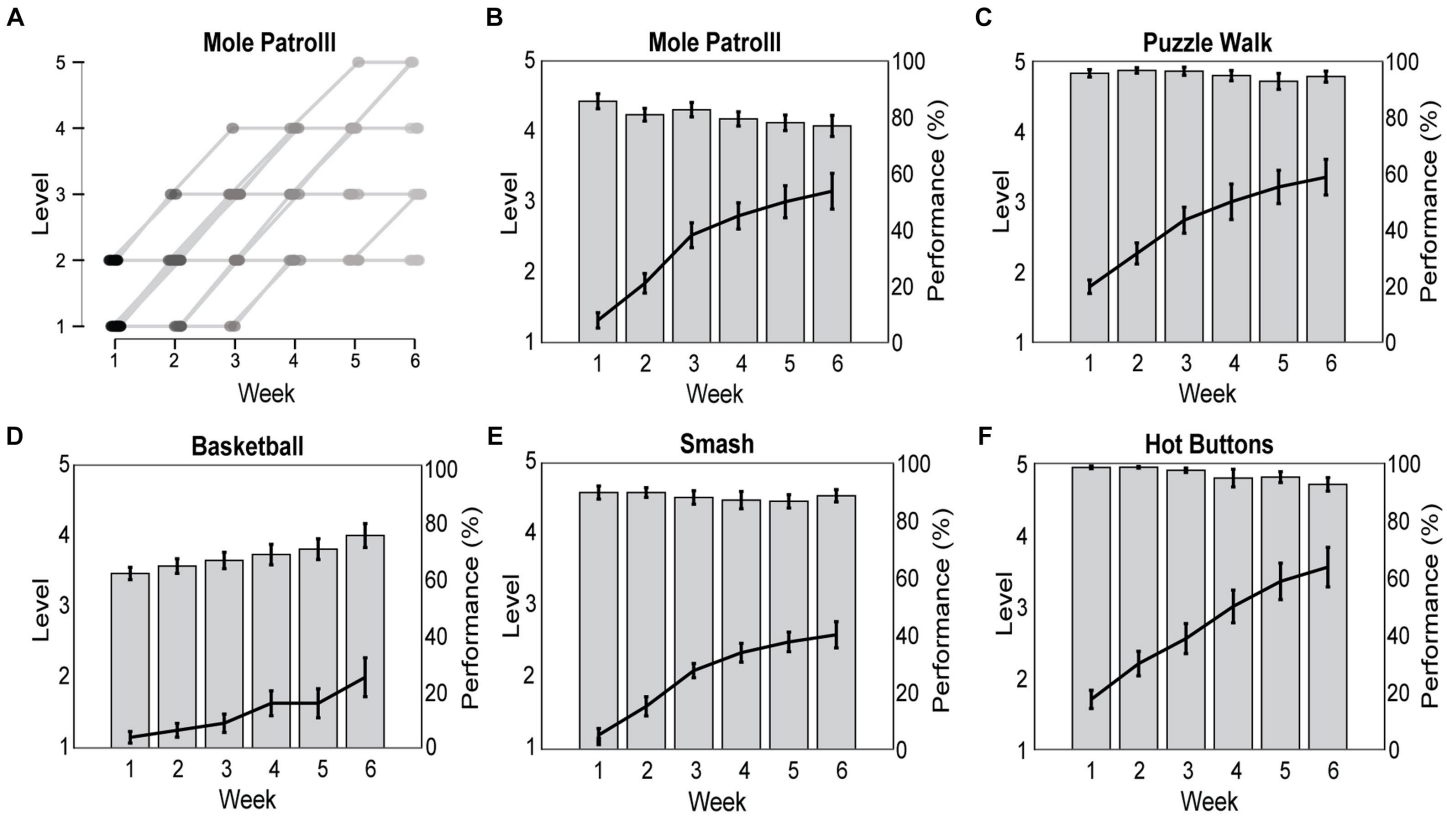
Reality DTx^®^ exergame-play levels were personalized to participants’ abilities and progression rates over the 6-week intervention **(A)** and prescribed in a progressive (i.e., significant increase in game-play levels over weeks; black lines) but achievable (high and non-varying game-play performance for all exergames but Basketball; gray bars) manner **(B–F)**.

Participants performed Reality DTx® with exergame-play levels tailored to their ability. There was a considerable variation in exergame-play level (Figure 6A, illustrated for Mole Patrolll), suggesting a successful personalization of the varying abilities and progression profiles of our participants. For the 20 participants completing the Reality DTx® intervention, Reality DTx® was a progressive-but-achievable intervention (Figures 6B–F), with exergame-play levels varying significantly over weeks for all exergames (*χ^2^*(5) > 32.321, *p* < 0.001), with significant linear contrasts indicating that for all exergames the levels increased proportionally over weeks (all *t*(df) > 5.840, *p* < 0.001). This progression in exergame levels did not differ significantly between groups. Exergame-performance scores were overall high-but-submaximal and did not vary systematically over weeks, except for basketball (*F*(2.29,39.00) = 10.417, *p* < 0.001), showing a proportional improvement in performance over weeks (*t*(85) = 7.128, *p* < 0.001, Figure 6D). Exergame performance did not differ significantly between groups.

#### 3.2.4 User experience

##### 3.2.4.1 Prescription lenses

All but one participant randomized to the ML2 group did not require prescription lenses to train with Reality DTx®, even though all ML2 participants used prescription (reading) spectacles or lenses in daily life. For pragmatic reasons, this participant with a prescription of +2.25 was moved to the HL2 group so that his spectacles could be worn during the intervention (i.e., to prevent delays and costs associated with ordering special lenses not part of the standard lens kit).

##### 3.2.4.2 Technical issues

The HL2 group participants reported predominantly issues related to shifts in or loss of the spatial map of the safe training area (with one dropout due to frustration with technical issues) and limited AR field of view. The ML2 group participants reported predominantly issues related to hand tracking (affecting interaction with menus Smash!, and Hot Buttons) and Wi-Fi connection. Such technical issues experienced during the intervention were categorized into issues that did or did not prevent participants from adhering to the prescribed intervention (Supplementary material S3). In only 10 of the 131 prescribed training weeks, more than 2 days per week were lost due to technical issues. These issues were solvable by participants themselves, by researchers visiting participants, or remotely through a telephone call.

##### 3.2.4.3 User Experience Questionnaire (UEQ)

**Figure 7:**
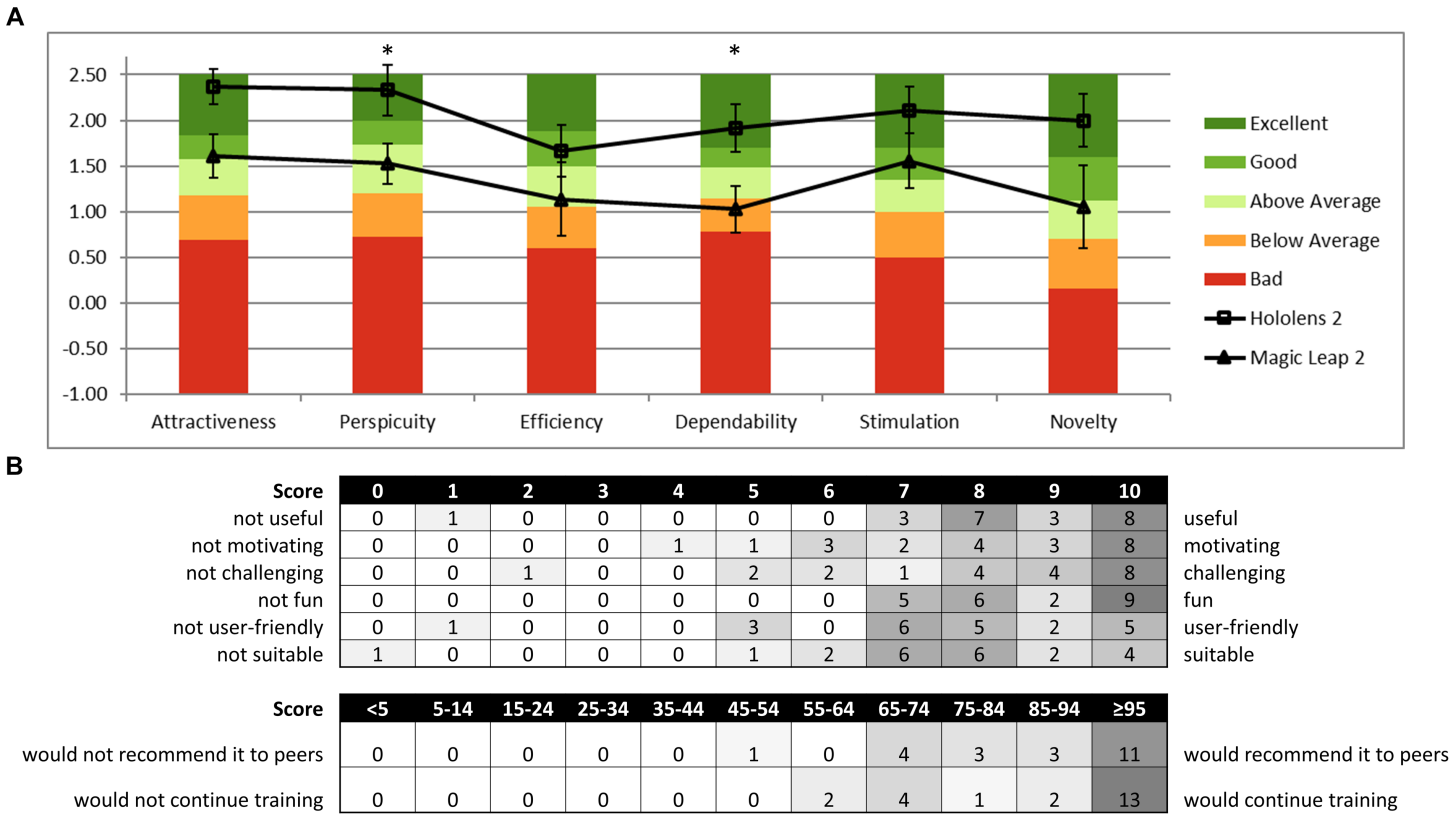
Reality DTx^®^ user experience and acceptance. **(A)** HL2 and ML2 group mean scores on the six domains of the User Experience Questionnaire (UEQ) relative to the benchmark scores of the questionnaire [*p < 0.05; analyses were based on *n* = 18 as four cases were excluded for inconsistencies following UEQ analysis guidelines (29)] and **(B)** distribution of the acceptability evaluation questionnaire score.

Reality DTx® reached above-average scores for UEQ (29) subscales Efficiency and Dependability, good scores for Perspicuity and Novelty, and excellent scores for Attractiveness and Stimulation (Figure 7A). User experience seemed overall somewhat better for the HL2 group (Figure 7A), with significantly lower scores for the ML2 group on Perspicuity (*U* = 64, *p* < 0.05, *r*_rb_ = 0.580, *BF*_10_ = 1.365) and Dependability (*t*(16) = 2.473, *p* < 0.05, *d* = 1.166, *BF*_10_ = 2.735) and borderline-significant lower scores for Attractiveness (*U* = 63, *p* = 0.051, *r*_rb_ = 0.556, *BF*_10_ = 1.615).

##### 3.2.4.4 Acceptability questions

Figure 7B depicts the score distribution on the acceptability evaluation Likert-scale questions, indicating that overall Reality DTx® was a well-accepted intervention. Participants scored the training as useful (8.4/10), motivating (8.2/10), challenging (8.1/10), fun (8.7/10), user-friendly (7.5/10), and suitable for improving gait and balance (7.5/10). On the question of how participants would feel if we stopped developing Reality DTx®, 17 of 22 participants indicated that they would be very disappointed, 5 of 22 indicated that they would be somewhat disappointed, and 0 of 22 indicated not to feel disappointed.

### 3.3 Potential efficacy

We conducted a 2 (Group) × 3 (Time) mixed ANOVA on outcomes of gait, balance, and walking-adaptability fall-risk indicators. We focussed on the main effects of Time, as effects with Group were generally not significant, except when explicitly mentioned (full statistics in Supplementary material S4).

#### 3.3.1 Clinical gait-and-balance tests

For Timed Up & Go test (TUG), 10 Meter Walk Test (10MWT), and Five Times Sit to Stand Test (FTSTS), a significant main effect of Time was observed (Table 3). For TUG, both inverse Helmert contrasts were significant, revealing that test completion times decreased from t0 to t1 and then decreased further at t2. For 10MWT, only the first and for FTSTS, only the second inverse Helmert contrast was significant, indicating improvements in completion times during the waitlist and after the intervention, respectively. Mini Balance Evaluation Systems Test (Mini-BESTest), MDS-UPDRS III, and Lindop Parkinson’s Physiotherapy Assessment Scale (LPAS) did not vary significantly with Time.

**Table 3:** Main effects of time and, when significant, their contrasts.

	t0	t1	t2	Main effect of time				First inverse Helmert contrast (t1-t0)			Second inverse Helmert contrast (t2-t1, t0)		
	M ± SD	M ± SD	M ± SD	F(df)*	*p*	*η_p_^2^*	*BF_10_*	*t*	*p*	Δt1-t0	*t*	*p*	Δt2-t1, t0
Clinical gait-and-balance test													
TUG (s)	11.65 ± 4.26	10.91 ± 3.98	10.39 ± 3.86	***F*(1.496,25.434)=6.084**	**0.012**	**0.264**	**8.339**	***t*(34) = −2.206**	**0.034**	**−0.80 ± 0.36**	***t*(34) = −2.703**	**0.011**	**−0.85 ± 0.31**
FTSTS (s)	16.85 ± 7.37	16.18 ± 6.11	13.46 ± 5.75	***F*(2,34) = 3.349**	**0.047**	**0.165**	**1.896**	*t*(34) = −0.347	0.731	−0.46 ± 1.34	***t*(34) = −2.565**	**0.015**	**−2.97 ± 1.16**
10MWT (s)	9.13 ± 1.97	8.51 ± 1.20	8.40 ± 1.33	***F*(2,34) = 5.216**	**0.011**	**0.235**	**6.788**	***t*(34) = −2.612**	**0.013**	**−0.62 ± 0.24**	*t*(34) = −1.900	0.066	−0.39 ± 0.21
Mini-BESTest	22.00 ± 3.71	22.16 ± 2.97	22.58 ± 3.95	*F*(2,34) =0.362	0.699	0.021	0.221	NA			NA		
MDS-UPDRS III	31.05 ± 11.31	31.63 ± 11.63	32.90 ± 10.77	*F*(2,34) =0.95 7	0.394	0.053	0.302	NA			NA		
LPAS	17.21 ± 1.55	17.42 ± 1.12	17.53 ± 1.22	*F*(2,34) =0.993	0.381	0.055	0.260	NA			NA		
Gait characteristics instrumented 10MWT													
Walking speed (cm/s)	113.86 ± 20.48	119.61 ± 16.50	121.71 ± 16.95	***F*(2,34) = 5.425**	**0.009**	**0.242**	**8.467**	***t*(34) = 2.400**	**0.022**	**5.64 ± 2.35**	***t*(34) = 2.256**	**0.031**	**4.59 ± 2.03**
Step length (cm)	65.74 ± 11.21	68.21 ± 10.41	68.70 ± 10.72	***F*(2,34) = 4.889**	**0.014**	**0.223**	**5.950**	***t*(34) = 2.473**	**0.019**	**2.43 ± 0.98**	*t*(34) = 1.914	0.064	1.63 ± 0.85
Step width (cm)	11.13 ± 3.89	10.83 ± 3.43	10.76 ± 3.88	*F*(2,34) =0.269	0.766	0.016	0.191	NA			NA		
Cadence (steps/min)	108.28 ± 10.37	110.08 ± 8.63	110.36 ± 8.80	*F*(2,34) =1.479	0.242	0.080	0.521	NA			NA		
Walking adaptability: obstacle avoidance													
Walking speed (cm/s)	104.44 ± 23.63	107.67 ± 17.72	113.28 ± 19.57	***F*(2,32) = 3.347**	**0.048**	**0.173**	**1.800**	*t*(32) = 0.985	0.332	3.13 ± 3.18	***t*(32) = 2.392**	**0.023**	**6.58 ± 2.75**
Success rate (%)	69.17 ± 31.59	66.11 ± 36.64	62.78 ± 37.39	*F*(2,32) = 0.560	0.577	0.034	1.154	NA			NA		
Margins (cm)	11.61 ± 6.10	12.07 ± 6.10	13.78 ± 5.18	*F*(2,32) = 2.410	0.106	0.131	0.957	NA			NA		
Walking adaptability: goal-directed stepping													
Normalized walking speed (%)	77.22 ± 21.13	81.83 ± 20.22	85.09 ± 18.57	***F*(2,32) = 3.671**	**0.037**	**0.187**	**2.321**	*t*(32) = 1.609	0.117	4.48 ± 2.78	***t*(32) = 2.180**	**0.037**	**5.25 ± 2.41**
Stepping accuracy (cm)	4.48 ± 1.39	4.13 ± 0.99	4.61 ± 1.37	*F*(2,32) = 2.024	0.149	0.112	0.570	NA			NA		
Walking adaptability: tandem walking													
Walking speed (cm/s)	82.89 ± 29.00	90.02 ± 22.53	98.22 ± 22.64	***F*(2,30) = 3.367**	**0.048**	**0.183**	**2.430**	*t*(30) = 1.257	0.219	6.72 ± 5.35	***t*(30) = 2.270**	**0.031**	**10.51 ± 4.63**
Sway (cm)	4.24 ± 1.47	3.83 ± 1.19	3.63 ± 1.36	*F*(2,30) = 2.244	0.124	0.130	0.883	NA			NA		
Walking adaptability: half-turns													
Turning time (s)	1.95 ± 0.82	1.78 ± 0.82	1.51 ± 0.47	***F*(1.321,21.144)** **= 4.133**	**0.045**	**0.205**	**1.553**	*t*(32) = −1.276	0.211	−0.21 ± 0.16	***t*(32) = −2.577**	**0.015**	**−0.36 ± 0.14**
Success rate (%)	27.78 ± 30.79	27.78 ± 35.24	27.78 ± 30.79	*F*(2,32) = 0.023	0.977	0.001	0.143	NA			NA		

*The assumption of sphericity was checked according to Girden (36). If Greenhouse–Geisser’s epsilon exceeded 0.75, the Huynh–Feldt degrees of freedom (df) correction was applied; otherwise, the Greenhouse–Geisser correction was used.

Bold values are significant analyses.

#### 3.3.2 Gait parameters

We quantified key gait characteristics during the instrumented 10MWT. For walking speed and step length, significant main effects of Time were observed (Table 3). Speed and step lengths increased from t0 to t1, and walking speed improved further at t2 after the Reality DTx® intervention. Step width and cadence did not vary with Time.

#### 3.3.3 Walking adaptability

Participants’ walking adaptability, a targeted marker for fall risk (26), improved after the Reality DTx® intervention, that is, at t2, participants completed the obstacle-avoidance, goal-directed stepping, tandem walking, and time-pressured half-turn tasks significantly faster than before, as reflected by significantly faster (normalized) walking speeds and turning times after the Reality DTx® intervention (Table 3), without negatively affecting walking-adaptability performance indicators such as obstacle-avoidance success rates and stepping accuracy (i.e., no effects of Time on walking-adaptability performance indicators; Table 3).

#### 3.3.4 Patient-reported outcome measures

For the questionnaires only a significant main effect of Time (*F*(2,36) = 3.309, *p* = 0.048, *η_p_^2^* = 0.155) was observed for FES-I, with a slightly but significantly 2.53 ± 1.22 higher FES-I score at t1 than at t0 (*t*(36) = 2.076, *p* = 0.045). Furthermore, a significant main effect of Group (*F*(1,18) = 5.224, *p* = 0.035, *η_p_^2^* = 0.225) was observed for NFOGQ, with a 6.88 ± 3.01 higher score for the ML2 (with 7/9 freezers) group compared to the HL2 (4/11 freezers) group.

## 4 Discussion

In this waitlist-controlled clinical feasibility study, we evaluated a home-based gait-and-balance exergaming intervention (Reality DTx^®^), a digital therapeutics program that was specifically designed for pwPD and uniquely administered through state-of-the-art AR glasses. Next, we discuss the findings in terms of their feasibility (safety, adherence, and user experience) and potential efficacy for improving gait, balance, and walking-adaptability fall-risk indicators.

### 4.1 Feasibility: Reality DTx® is a safe, adherable, well-accepted, and usable intervention

A key feasibility aspect of new therapy interventions is safety, which seems especially relevant for Reality DTx® given its unsupervised remote delivery in an intrinsically high fall-risk population. We found that Reality DTx® was safe (no falls, only four near falls in >15,000 active minutes of gait-and-balance exergaming) with limited adverse events in relevant prespecified (30, 31) domains (e.g., some reports of dizziness, no eyestrain, and two headaches). We learned that exergame settings could be adjusted to prevent adverse events like dizziness, thereby further improving safety. For example, lower Smash! exergame levels yielded high turning rates, which may cause dizziness (i.e., 8/15 dizziness reports were attributed to turning), which can be remedied by lowering induced turning rates (e.g., demanding more punches and increasing inter-plinth distances). Exergame settings were also adjusted to tailor the physical load of the Reality DTx® intervention (according to FITT principles) to the participant’s physical capacity; still, some adverse events in the ‘other’ class were reported (such as re-occurring injuries).

A second important feasibility aspect is adherence. Our participants were able to exercise independently at home with Reality DTx®, with 104% session adherence, which is high compared to known adherence rates for home-based exercise interventions [e.g., 84% in (32)]. This is an encouraging finding considering the high-dose default prescription of 30 active minutes/session for five sessions/week for 6 weeks (i.e., note that total session duration was always longer than the prescribed active minutes due to, e.g., switching or rests between exergames). Participants performed slightly fewer active minutes than prescribed (88% active-minute/session adherence). Still, this led to a high number of repetitions and a high dose of sit-to-stands/squats, functional reaches, and meters walked compared to other home-based interventions (33). For some participants, the default 30 active minutes/session was adjusted over weeks to tailor it, for example, to their physical capacity or time constraints. This again emphasizes how important remote monitoring and shared decision-making are for prescribing a progressive-but-achievable intervention as will be discussed next.

Reality DTx® was not only remotely monitored for adherence, but also for exergame performance. Reality DTx® was intended as a progressive-but-achievable intervention, balancing task demands and capacity (not too easy to prevent boredom and not too difficult to prevent demotivation). We found that exergame levels indeed progressed significantly over weeks, with participant-specific exergame levels and progression rates (i.e., tailored treatment), whereas the consistently high-but-submaximal exergame-performance scores over the weeks indicated that the intervention was achievable. Reality DTx® thus seemed to comply with the intended progressive-but-achievable principle, which is a prerequisite for reaching an intrinsically rewarding and highly engaged ‘flow state,’ associated with exceptional performance and potentially increased long-term adherence (34, 35).

The third key feasibility aspect is the acceptance and usability of interventions. Overall, Reality DTx® was a well-accepted intervention. User experience scores for Reality DTx® were excellent on UEQ domains Stimulation and Attractiveness, good on Novelty and Perspicuity, and above average on Dependability and Efficiency compared to other established products [i.e., UEQ benchmark scores (29)]. Note that we found superior Dependability (‘*Does the user feel in control of the interaction? Is it secure and predictable?*’) and Perspicuity (‘*Is it easy to get familiar with the product and learn how to use it?*’) scores for HL2 than for ML2 AR glasses, most likely due to the at-that-time poorer hand tracking of ML2, as was also more often reported as a technical issue by the ML2 group participants. We cannot conclude on a clear winner in terms of AR glasses superiority (our secondary objective) as both AR glasses had their distinct advantages and disadvantages for different feasibility aspects (e.g., use with own glasses better for HL2, AR field of view better for ML2, hand tracking superior for HL2, and spatial mapping better for ML2). Furthermore, rapid progress in software developments for AR glasses continues to improve usability and performance with each update (e.g., ML2 hand tracking has been improved considerably with a recent update), so future studies will likely not be hindered by the technical issues and limitations we experienced with specific AR glasses. The same holds for issues related to the Reality DTx® digital therapeutics platform (e.g., connectivity, mapping, and bugs), which were reported to Strolll Limited for further development and improvement.

All in all, Reality DTx® is a safe, adherable, well-accepted, and usable intervention, and its feasibility is likely to improve even further based on the learnings of this study.

### 4.2 Potential efficacy: Reality DTx^®^ is promising for improving targeted fall-risk indicators

The potential efficacy of Reality DTx® for improving gait, balance, and fall risk was evaluated comprehensively, using outcomes covering standard clinical tests, gait characteristics, and advanced walking-adaptability assessments as targeted fall-risk indicators.

Concerning standard clinical tests, significant intervention effects were observed for TUG and FTSTS, suggesting improvements in functional mobility, lower limb strength, and dynamic balance (7, 37–40) in a relatively high-functioning (i.e., HY2-2.5) group of pwPD recruited from the general public. The significant post-intervention TUG improvement of 0.85 ± 0.31 s against a ~ 11 s baseline group TUG-time was smaller than the 1.63 s minimal detectable change (MDC) (41), whereas the significant post-intervention FTSTS improvement of 2.97 ± 1.16 s against a ~ 16 s baseline group FTSTS-time was substantially greater than the 1.66 s MDC [i.e., derived from the standard error of measurement score of 0.6 s in (42) and greater than the 2.5 s minimal clinically importance difference in Spagnuolo et al. (43)]. TUG and 10MWT were prone to small waitlist effects (i.e., significant improvements during the waitlist period), reminiscent of a Hawthorne effect (44, 45) as observed before [e.g., (46)] or due to learning/familiarization with the tests or test setting. Other standard clinical tests did not vary systematically (Mini-BESTest and LPAS), probably hindered by ceiling effects [i.e., ≥20% of the sample received the maximum score on all Mini-BESTest subscales, except for reactive postural control, and on the LPAS subscale scores (47)]. For the MDS-UPDRS III, an absence of effect may be explained by the minor emphasis on gait and balance and the shorter-than-recommended 12-week training period for achieving clinically meaningful improvements in the severity of motor systems [as measured with MDS-UPDRS III (48)].

Concerning the assessments with the Interactive Walkway (Figure 3), we found an improved post-intervention walking speed for gait characteristics and profound intervention effects for adaptive walking, with faster test completion times without negatively affecting performance. These findings were robust (i.e., without any waitlist-period effects that hampered some of the standard clinical tests and gait-characteristic outcomes), suggesting targeted effects of Reality DTx® for improving walking-adaptability fall-risk indicators (26). This is encouraging as Reality DTx® exergames were designed to explicitly target this construct. Note that walking adaptability is not well captured with standard clinical tests (26). The observed targeted improvements in walking adaptability are promising as they tentatively lower one’s fall risk (26) as may be evaluated in future Reality DTx® effect studies.

All in all, Reality DTx® seems promising for improving aspects of gait and balance, in particular on lower limb strength, dynamic balance (i.e., FTSTS), and walking-adaptability as fall-risk indicators (26, 37).

### 4.3 Recommendation for future research

Above-discussed results on the feasibility and potential efficacy of Reality DTx® warrant future controlled effect studies, for which we recommend:

- Changing inclusion criteria: We learned that Reality DTx® was a feasible unsupervised at-home intervention for participants with HY2 and HY2.5. Our inclusion criteria were HY2-4, but we excluded two participants with HY3 at t0 as their fall risk was deemed too high for unsupervised exergame, while HY4 did not enter the study at all. We recommend broadening inclusion to HY1. This is relevant as gait-and-balance impairments and fall risk are already present from an early stage (1) and people in this stage may benefit from targeted gait-and-balance interventions. People with PwPD with higher HY stages with increased fall risk could use Reality DTx® first under supervision in the clinic (see ii) and/or tailored to their ability (e.g., see iii). These recommendations are implemented in the indications by Strolll Limited.
- Combining clinical and at-home exergaming settings: With this study, we were quite ambitious by starting home-based exergaming after limited familiarization and instruction time. By delivering Reality DTx® in a hybrid form, starting in the clinical pathway for some sessions before taking it home, more time for instructions, familiarization, and evaluation of safety is available. This tentatively improves the confidence of inclusion/exclusion of people with HY3 and enables supervised in-clinic exergaming scenarios for people with HY4 (see iii);
- Extending the number of exergames: To target other aspects of motor and/or cognitive impairments [e.g., dual-tasking (14, 23, 24, 49)], to include those at higher HY stages with tailored game-play settings (e.g., playing when seated), and to increase long-term adherence (e.g., playing the same five exergames may become less engaging or motivating over a longer period);
- Considering changing outcome measures: The observed intervention effects of Reality DTx® were convincing for improving targeted fall-risk indicators associated with walking adaptability, fitting the nature of the exergames. Hence, future studies may consider designing effect studies targeting fall risk or prospective falls as outcome measures, which seems relevant given the high fall incidence in this population. Future studies may also add health-economic outcomes as Reality DTx® may contribute to extending the number of (unsupervised) rehabilitation exercise hours while lowering the burden on healthcare professionals and increasing accessibility and adherence to treatment, in the convenience of users’ own homes and time, instead of supervised in the clinic;
- Extending intervention interval: We used a 6-week intervention period, which may be on the lower end of the guideline recommendations (10, 12, 50). Participants were positive about continuing with Reality DTx® after the 6-week intervention (Figure 7).

## 5 Conclusion

We found that the remotely prescribed, monitored, and tailored Reality DTx® intervention was feasible: It is safe for use at home, adherable, progressive-but-achievable, well-accepted, and usable. Reality DTx® was potentially effective for improving gait and balance, in particular for lower limb strength, dynamic balance, and walking adaptability as indicators of reduced falls fall risk. Future controlled effect studies with this feasible and potentially effective Reality DTx® digital therapeutics platform are thus warranted.

## Data availability statement

The original contributions presented in the study are included in the article/Supplementary material (See Supplementary material S5), further inquiries can be directed to the corresponding author.

## Ethics statement

The studies involving humans were approved by Medical Research Ethics Committees United, The Netherlands. The studies were conducted in accordance with the local legislation and institutional requirements. The participants provided their written informed consent to participate in this study. Written informed consent was obtained from the individual(s) for the publication of any potentially identifiable images or data included in this article.

## Author contributions

LH: Investigation, Writing – original draft, Writing – review & editing. DG: Conceptualization, Investigation, Writing – original draft, Writing – review & editing. EH: Investigation, Writing – original draft, Writing – review & editing. JN: Conceptualization, Supervision, Writing – original draft, Writing – review & editing. MR: Conceptualization, Funding acquisition, Supervision, Writing – original draft, Writing – review & editing.

## Supplementary material

Data Sheet 1 Supplementary Material Tables S1-4.

Data Sheet 2 Supplementary Material Table S5.
