# Supplementary material for: Remotely prescribed, monitored, and tailored home-based gait-and-balance exergaming using augmented reality glasses: a clinical feasibility study in people with Parkinson’s disease
Source: Front Neurol. 2024 May 30;15:1373740. doi: 10.3389/fneur.2024.1373740 (PMC11172158; doi:10.3389/fneur.2024.1373740)
Supplement: Data Sheet 1 — Supplementary Material Tables S1-4. [file Data_Sheet_1.doc]

Supplementary Material

**Table S1. Description of the five AR gait-and-balance Reality DTx^®^ exergames, including available game statistics.**

| **Reality DTx^®^ game** | **Description of the game** | **Game statistics** | **Game levels** |
| --- | --- | --- | --- |
| **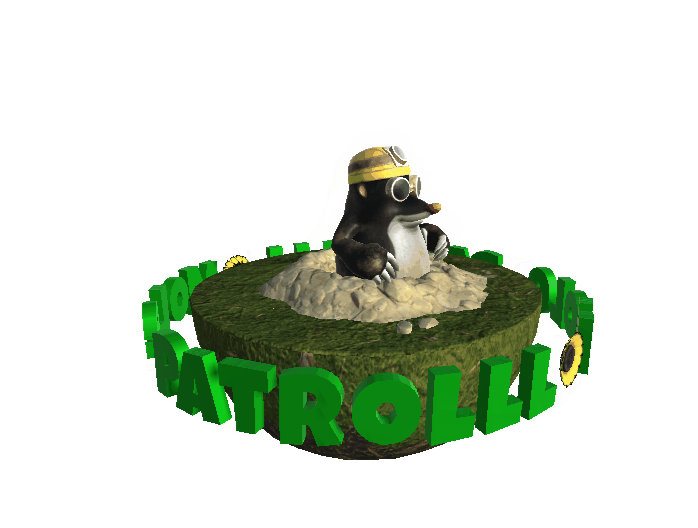**  **Mole Patrolll** | A goal-directed walking exergame to train gait initiation, dynamic balance, turning, stopping and strength (when performed in squat mode). The goal is to stomp as many moles as possible by scanning the room, spotting where they appear, and stomping on them either with both feet or squatting on them (a game-mode setting) before they disappear. Mole appearance duration reduces over difficulty levels to make the game more challenging. | **In-game feedback** Number of moles caught, distance walked  **Post-game feedback** Number of moles caught  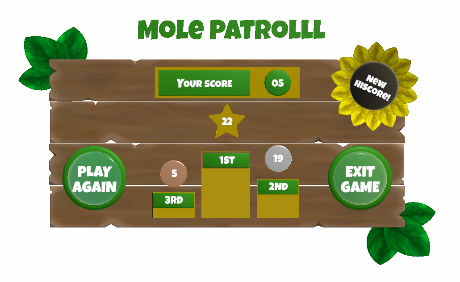 **Web portal feedback** Prescribed and active minutes Meters walked | **The time the mole is out of the molehill:**  Level 1:  6o seconds p/m  Level 2: 10 seconds p/m  Level 3:  7.5 seconds p/m  Level 4: 4 seconds p/m  Level 5:  1 second p/m |
| **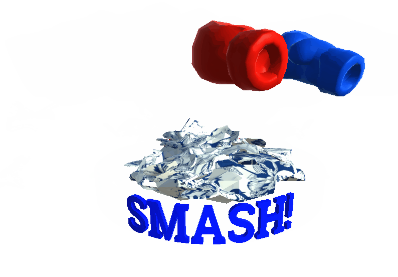**  **Smash!** | A boxing exergame to train gait, dynamic balance, weight shifting and turning. The goal is to smash as many items as possible from two plinths as they appear, demanding alternate left-right punches to promote weight shifting, with available items alternating between the plinths to promote walking and turning. The distance between the plinths is adjustable (ranging from 2 to 10 meters) and so is the number of required punches before the items drop from the plinth (ranging from 2-20 over difficulty levels) to make the game more challenging. | **In-game feedback** Number of items smashed, number of prescribed and performed punches  **Post-game feedback** Number of items smashed  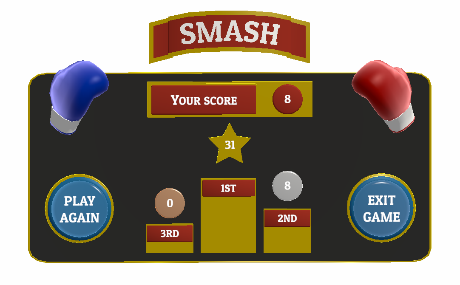  **Web portal feedback** Prescribed and active minutes Meters walked Number of functional reaches | **The number of punches for the items to fall off the plinths:**  Level 1: 2 punches  Level 2: 5 punches  Level 3: 10 punches  Level 4: 15 punches  Level 5:  20 punches |
| **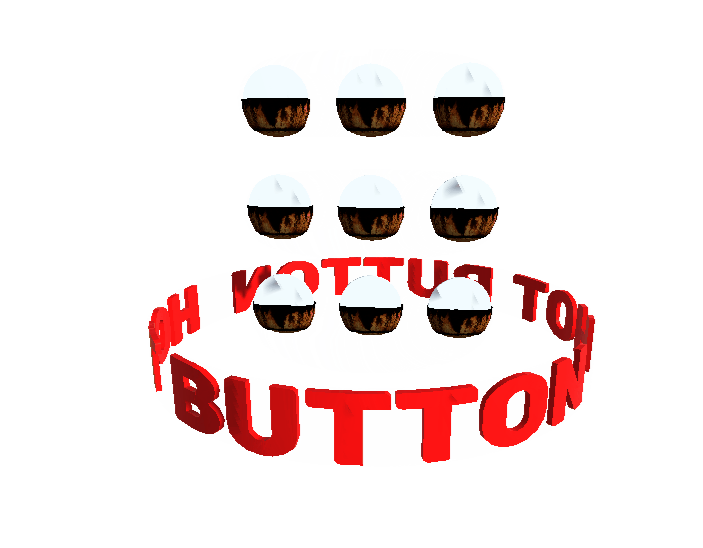**  **Hot Buttons** | A dynamic reaching exergame to train functional reaching, reaction time and dynamic balance. The goal is to press the button that lights up as quickly as possible before it disappears. Available buttons are presented in rows of three, stacked vertically totaling either 3, 6 or 9 buttons, dependent on the mode. The reach distance is adjustable (40-90cm) and feet positioning is controlled to avoid cheating. Dependent on the mode, the participant presses the buttons either with the left, right or both hands at random. The appearance duration of the light-up buttons decreases over difficulty levels to make the game more challenging. | **In-game feedback** Number of buttons hit and streaks (i.e., hitting two or more buttons in a row with the prescribed hand)  **Post-game feedback** Number of buttons hit. Bonus points for streaks which add up dependent on the number of buttons hit in a row. You lose the streak when hitting a button with the wrong hand.  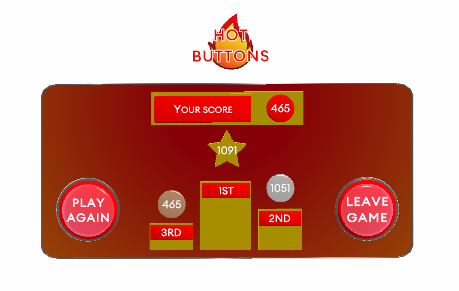  **Web portal feedback** Prescribed and active minutes Number of functional reaches | **The time between buttons lighting up:**  Level 1: Timeout after 60 seconds  Level 2: Timeout after 15 seconds  Level 3: Timeout after 8 seconds  Level 4: Timeout after 3 seconds  Level 5: Timeout after 1.5 seconds |
| **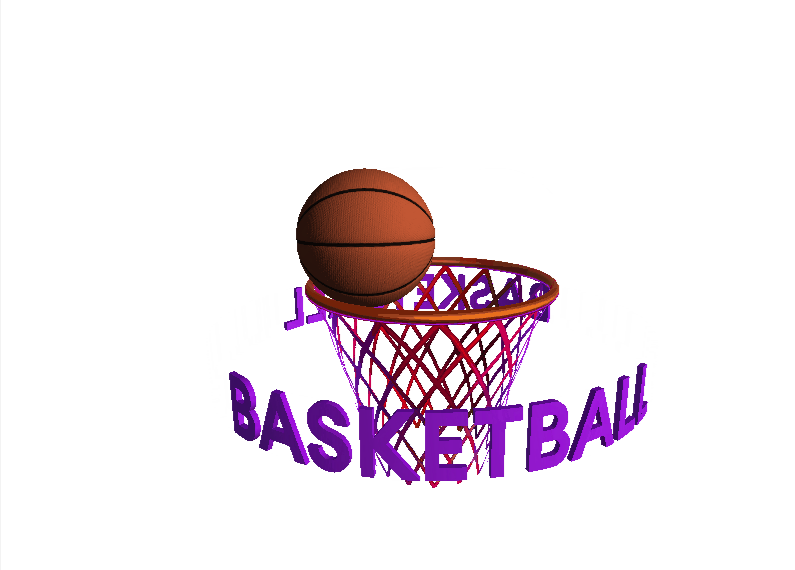**  **Basketballl** | A sit-to-stand exergame to train dynamic balance and lower-limb muscle strength. The goal is to score as many points as possible by completing sit-to-stand or squat-to-stand movements (a game-mode setting) to spawn a set of three basketballs, and throw them into the hoop. The number of required sit-to-stand or squat-to-stand movements to earn basketballs increases over difficulty levels to make the game more challenging. | **In-game feedback** Number of prescribed and performed sit- or squat-to-stands, number of basketballs scored  **Post-game feedback** Number of sit- or squat-to-stands  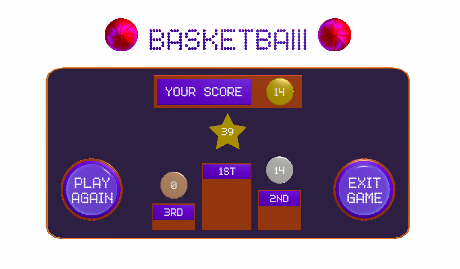  **Web portal feedback** Prescribed and active minutes Number of sit-to-stands | **The number of sit-to-stand or squats:**  Level 1:  3  Level 2: 6  Level 3: 9  Level 4: 12  Level 5: 15 |
| **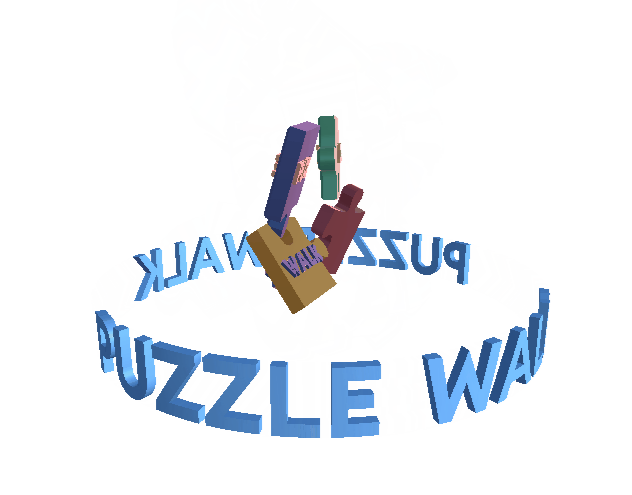**  **Puzzle Walk** | A goal-directed walking exergame to train gait, dynamic balance, turning, stopping and functional reaching. The goal is to find puzzle pieces in the room, pick them up by reaching the hand to them and place them on the easel to complete the puzzle before the time runs out. The required reaching height to collect the puzzle pieces is adjustable (selection from high, hip-level, knee-level or floor-level reaches). The number of puzzle pieces to complete the puzzle varies over difficulty levels to make the game more challenging. | **In-game feedback** Number of prescribed and collected puzzle pieces, time left  **Post-game feedback** Number of collected puzzle pieces within the set game duration (bonus points for every second left on the clock)  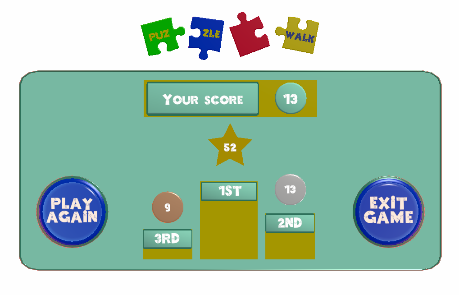  **Web portal feedback** Prescribed and active minutes Meters walked Number of functional reaches | **The number of puzzle pieces:**  Level 1: 9 pieces  Level 2: 16 pieces  Level 3: 24 pieces  Level 4: 30 pieces  Level 5: 48 pieces |

| **Table S2.** Data-collection methods, outcome measures and time points of data collection for aspects of feasibility (safety, adherence, user experience) and potential efficacy for improving gait and balance (including walking adaptability). | | |
| --- | --- | --- |
| **Data collection** | **Outcome measure** | **Timepoint of data collection** |
| **Feasibility** |  |  |
| ***Safety:***  **Weekly telephone calls** | **Number of falls, near falls and adverse events (dizziness, headache, eyestrain, other (39-41))** | **t1 – t2** |
| ***Adherence:***  **-** | **Drop-out rate** | **t1 – t2** |
| **Reality DTx^®^ web portal** | **Session adherence (%)**  **Active-minutes/session adherence (%)** | **t1 – t2** |
| ***Game performance:***  **Reality DTx^®^ web portal** | **Mole Patrolll: Number of moles caught / Total number of moles spawned**  **Smash!: Number of vases smashed / Total number of vases spawned**  **Basketballl: Number of sit-to-stand movements / Target number of sit-to-stands movements per minute**  **Hot Buttons: Number of buttons pressed / Total number of buttons lit up**  **Puzzle walk: Number of pieces / Total pieces to be collected** | **t1 – t2** |
| ***User* *experience:*** | |  |
| **User Experience Questionnaire (UEQ)** | **Contrasting attributes (1-7 Likert scale) related to Attractiveness, Perspicuity, Efficiency, Dependability, Stimulation and Novelty** | **t2** |
| **Acceptability evaluation questionnaire** | **Scores (between 0-10 and 0-100%) on various acceptability questions** | **t2** |
| **Weekly telephone calls** | **Number of reported technical issues** | **t1 – t2** |
| **Potential efficacy** |  |  |
| ***(Adaptive) gait-and-balance tests:*** |  | **t0, t1, t2** |
| **Mini Balance Evaluation Systems Test (Mini-BESTest) (42)** | **Total score (0-28)** |  |
| **Timed Up-and-Go test (TUG) (25, 27)** | **Completion time (s)** |  |
| **Five Times Sit-to-Stand Test (FTSTS) (24, 26)** | **Completion time (s)** |  |
| **Lindop Parkinson's Physiotherapy Assessment Scale (LPAS) (43)** | **Total score of gait mobility subscale (0-18)** |  |
| **Interactive Walkway* (16)**  **10-meter walking task (as measured by the Interactive Walkway (16))** | **Obstacle avoidance: Outcome measures were walking speed (cm/s), success rate (%) and obstacle-avoidance margins (cm)**  **Goal-directed stepping: Outcome measures were normalized walking speed (%) and stepping accuracy (cm)**  **Tandem walking: Outcome measures were walking speed (cm/s) and mediolateral sway (cm)**  **Half turns: Outcome measures were turning time (s) and success rate (%)**  **Walking speed (cm/s), step length (cm), step width (cm) and cadence (steps/min)** |  |
| ***Patient-reported outcome measures*** |  | **t0, t1, t2** |
| **Movement Disorders Society Unified Parkinson Disease Rating Scale – motor score (MDS-UPDRS III) (44)** | **Total score (0 – 132); higher scores mean more lower motor functioning** |  |
| **Physical Activity Scale for the Elderly (PASE) (45)** | **Total score (0-400); higher scores mean a higher level of physical activity** |  |
| **New Freezing of Gait Questionnaire (NFOGQ) (46)** | **Total score (0-28); lower scores mean less freezing of gait** |  |
| **Activities-Specific Balance Confidence Scale (ABC) (47)** | **Total score (0-100); higher scores mean more balance confidence** |  |
| **Falls Efficacy Scale International (FES-i) (48)** | **Total score (16-64); higher scores mean more fear of falling** |  |
| **Parkinson's Disease Questionnaire (PDQ-39) (49)** | **Total score (0-156); higher scores mean a lower experienced quality of life** |  |

*Note*. Session adherence = ratio of performed to prescribed number of exergaming sessions, Active-minutes/session adherence = ratio of performed number of sessions to performed active minutes during these sessions.
*Adaptive walking outcome measures were calculated as detailed in (16), with the addition of mediolateral sway during tandem walking (standard deviation of mediolateral spine-shoulder positions).

| **Table S3. Technical issues that did and did not prevent ML2 and HL2 participants from adhering to the prescribed training program (i.e., five sessions/week, 30 active minutes/session).** | | | | | | |  |
| --- | --- | --- | --- | --- | --- | --- | --- |
| **Categories of issues preventing participants from adhering to the training program:** | **Number of reported issues per group / total number of reported issues over 6 weeks** | | **Categories of issues not preventing participants to adhere to the training program:** | **Number of reported issues per group / total number of reported issues over 6 weeks (%)** | | |  |
|  | **ML2** | **HL2** |  | | **ML2** | **HL2** | |
| **1) The participant needs to make a new room scan but cannot do this independently** | **0/2** | **1/10** | **A) AR glasses suddenly switch off** | | **24/162**  **(14.8)** | **26/146**  **(17.8)** | |
| **2) Shifting of the digital spatial map of the training area. Guiding the participant to make a new room scan does not solve the issue** | **0/2** | **5/10** | **B) Limited field of view** | | **6/162**  **(3.7)** | **18/146 (12.3)** | |
| **3) Malfunctioning Wi-Fi connection preventing participants from logging in with their personal pin number)** | **2/2** | **2/10** | **C) Hand tracking issues (e.g., participants experience difficulty punching items with Smash! or pressing buttons)** | | **57/162 (35.2)** | **31/146 (21.2)** | |
| **4) Games do not show in the game menu because of communication issues (i.e., communication with the web portal or through Wi-Fi)** | **0/2** | **2/10** | **D) Communicational issues related to the training program data from the web portal** | | **4/162 (2.5)** | **18/146 (12.3)** | |
|  |  |  | **E) Connectivity issues related to Wi-Fi (i.e., participants cannot log in with their personal pin number)** | | **15/162 (9.3)** | **2/146 (1.4)** | |
|  |  |  | **F) Issues with shifting or loss of digital spatial map of the training area (due to this issue, a new digital room scan was sometimes required)** | | **29/162 (17.9)** | **41/146 (28.1)** | |
|  |  |  | **F) Issues with calibrating participant’s length, sitting height and arm length resulting in misplacement of digital targets in games (e.g., puzzle pieces)** | | **17/162 (10.5)** | **10/146 (6.8)** | |
|  |  |  | **G) Other hardware-related issues (e.g., difficulty in training under certain lighting circumstances)** | | **10/162**  **(6.2)** | **7/146 (4.8)** | |

***Note*. Some issues that did and did not prevent participants from adhering to the training program are related. These are: 1, 2 – F; 3, 4 – D, E.**

| **Table S4:** Group, time and interaction effects for (adaptive) gait-and-balance outcomes. | | | | | | | | | | | | | |
| --- | --- | --- | --- | --- | --- | --- | --- | --- | --- | --- | --- | --- | --- |
|  | **Group (HL2, ML2)** | | | | **Time (t0, t1, t2)** | | | | **Group-by-Time Interaction** | | | | |
|  | ***F**** | ***p*** | ***η_p_^2^*** | ***BF_10_*** | | ***F**** | ***p*** | ***η_p_^2^*** | ***BF_10_*** | ***F**** | ***P*** | ***η_p_^2^*** | ***BF_10_*** |
| ***Clinical gait and balance tests*** | | | | | | | | | | | | | |
| TUG | *F*(1,17)=0.446 | 0.513 | 0.026 | 0.706 | | ***F*(1.496,25.434) =6.084** | **0.012** | **0.264** | **8.339** | *F*(1.496,25.434) =1.100 | 0.331 | 0.061 | 0.421 |
| 5TSTS | *F*(1,17)=2.781 | 0.114 | 0.141 | 1.085 | | ***F*(2,34)=3.349** | **0.047** | **0.165** | **1.896** | *F*(2,34)=0.570 | 0.571 | 0.032 | 0.323 |
| 10MWT | *F*(1,17)=0.004 | 0.953 | <0.001 | 0.568 | | ***F*(2,34)=5.216** | **0.011** | **0.235** | **6.788** | *F*(2,34)=0.574 | 0.568 | 0.033 | 0.331 |
| Mini-BESTest | *F*(1,17)=0.131 | 0.722 | 0.008 | 0.595 | | *F*(2,34)=0.362 | 0.699 | 0.021 | 0.221 | *F*(2,34)=1.522 | 0.233 | 0.082 | 0.607 |
| MDS-UPDRS III | *F*(1,17)=0.022 | 0.883 | 0.001 | 0.667 | | *F*(2,34)=0.957 | 0.394 | 0.053 | 0.302 | *F*(2,34)=0.004 | 0.996 | <0.001 | 0.236 |
| LPAS | *F*(1,17)=0.001 | 0.973 | <0.001 | 0.504 | | *F*(2,34)=0.993 | 0.699 | 0.021 | 0.260 | *F*(2,34)=1.770 | 0.186 | 0.094 | 0.694 |
| **Gait characteristics instrumented 10MWT** | | | | | | | | | | | | | |
| Walking speed | *F*(1,17)=0.019 | 0.893 | 0.001 | 0.599 | | ***F*(2,34)=5.425** | **0.009** | **0.242** | **8.467** | *F*(2,34)=0.777 | 0.468 | 0.044 | 0.391 |
| Step length | *F*(1,17)=0.047 | 0.832 | 0.003 | 0.715 | | ***F*(2,34)=4.889** | **0.014** | **0.223** | **5.950** | *F*(2,34)=0.323 | 0.726 | 0.019 | 0.286 |
| Step width | *F*(1,17)=0.111 | 0.743 | 0.006 | 0.672 | | *F*(2,34)=0.269 | 0.766 | 0.016 | 0.191 | *F*(2,34)=0.832 | 0.444 | 0.047 | 0.400 |
| Cadence | *F*(1,17)=0.842 | 0.372 | 0.047 | 0.856 | | *F*(2,34)=1.479 | 0.242 | 0.080 | 0.521 | *F*(2,34)=1.172 | 0.322 | 0.064 | 0.481 |
| **Walking adaptability: obstacle avoidance** | | | | | | | | | | | | | |
| Walking speed | *F*(1,16)=0.821 | 0.378 | 0.049 | 0.697 | | ***F*(2,32)= 3.347** | **0.048** | **0.173** | **1.800** | *F*(2,32)=2.234 | 0.124 | 0.123 | 0.928 |
| Success rate | *F*(1,16)=3.034 | 0.101 | 0.159 | 1.154 | | *F*(2,32)=0.560 | 0.577 | 0.034 | 0.238 | *F*(2,32)=0.496 | 0.614 | 0.030 | 0.315 |
| Margins | *F*(1,16)=0.332 | 0.573 | 0.020 | 0.615 | | *F*(2,32)=2.410 | 0.106 | 0.131 | 0.971 | *F*(2,32)=0.476 | 0.626 | 0.029 | 0.316 |
| **Walking adaptability: goal-directed stepping** | | | | | | | | | | | | | |
| Normalized walking speed | *F*(1,16)=0.225 | 0.641 | 0.014 | 0.636 | | ***F*(2,32)=3.671** | **0.037** | **0.187** | **2.321** | *F*(2,32)=0.764 | 0.474 | 0.046 | 0.413 |
| Accuracy | *F*(1,16)=0.283 | 0.602 | 0.017 | 0.550 | | *F*(2,32)=2.024 | 0.149 | 0.112 | 0.570 | *F*(2,32)=0.518 | 0.601 | 0.031 | 0.333 |
| **Walking adaptability: tandem** | | | | | | | | | | | | | |
| Walking speed | *F*(1,15)=0.110 | 0.745 | 0.007 | 0.500 | | ***F*(2,30)=3.367** | **0.048** | **0.183** | **2.430** | *F*(2,30)=1.270 | 0.296 | 0.078 | 0.561 |
| Sway | *F*(1,15)=0.838 | 0.374 | 0.053 | 0.644 | | *F*(2,30)=2.244 | 0.124 | 0.130 | 0.883 | *F*(2,30)=1.025 | 0.371 | 0.064 | 0.458 |
| **Walking adaptability: half turns** | | | | | | | | | | | | | |
| Turning time | *F*(1,16)=1.299 | 0.271 | 0.075 | 0.671 | | ***F*(1.321,21.144) =4.133** | **0.045** | **0.205** | **1.553** | *F*(1.321,21.144) =2.503 | 0.121 | 0.135 | 0.982 |
| Success rate | *F*(1,16)=0.012 | 0.915 | <0.001 | 0.402 | | *F*(2,32)=0.023 | 0.977 | 0.001 | 0.143 | *F*(2,32)=1.877 | 0.169 | 0.105 | 1.224 |
| **The assumption of sphericity was checked according to Girden (55). If Greenhouse–Geisser’s epsilon exceeded 0.75, the Huynh-Feldt degrees of freedom (df) correction was applied; otherwise the Greenhouse–Geisser correction was used.* | | | | | | | | | | | | | |
